# Reactions in Nitroimidazole Triggered by Low-Energy (0**–**2 eV) Electrons: Methylation at N1-H Completely Blocks Reactivity**

**DOI:** 10.1002/anie.201407452

**Published:** 2014-09-15

**Authors:** Katrin Tanzer, Linda Feketeová, Benjamin Puschnigg, Paul Scheier, Eugen Illenberger, Stephan Denifl

**Affiliations:** Institut für Ionenphysik und Angewandte Physik and Center of Molecular Biosciences, Leopold Franzens Universität InnsbruckTechnikerstrasse 25, 6020 Innsbruck (Austria); ARC Centre of Excellence for Free Radical Chemistry and Biotechnology, School of Chemistry and Bio21 Institute of Molecular Science and Biotechnology, The University of Melbourne30 Flemington Road, Victoria 3010 (Australia); Institut für Chemie und Biochemie-Physikalische und Theoretische ChemiFreie Universität Berlin, Takustrasse 3, 14195 Berlin (Germany)

**Keywords:** gas-phase reactions, low-energy electrons, mass spectrometry, metastable compounds, reaction mechanisms

## Abstract

Low-energy electrons (LEEs) at energies of less than 2 eV effectively decompose 4-nitroimidazole (4NI) by dissociative electron attachment (DEA). The reactions include simple bond cleavages but also complex reactions involving multiple bond cleavages and formation of new molecules. Both simple and complex reactions are associated with pronounced sharp features in the anionic yields, which are interpreted as vibrational Feshbach resonances acting as effective doorways for DEA. The remarkably rich chemistry of 4NI is completely blocked in 1-methyl-4-nitroimidazole (Me4NI), that is, upon methylation of 4NI at the N1 site. These remarkable results have also implications for the development of nitroimidazole based radiosensitizers in tumor radiation therapy.

Low-energy electrons (LEEs) can induce remarkably selective reactions in molecules,[1,2] which is based on the initial formation of an intermediate metastable electron–molecule compound (M^.−#^; M=molecule). Such a transient negative ion can decompose into a neutral and negatively charged fragment (dissociative electron attachment, DEA; reaction (1 a)):

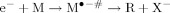
(1a)

DEA always competes with the loss of the extra electron recovering the neutral molecule, possibly in an excited state (autodetachment; reaction (1 b)):[1]

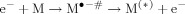
(1b)

In the DNA base thymine for example, the loss of a neutral H atom via DEA at electron energies below 3 eV is bond-selective (only the two N=H bonds are involved),[3] which can be made even site-selective by properly tuning the electron energy; that is, at electron energies below 1.2 eV, the loss of H exclusively involves the N1 site.[4] Furthermore, it has been demonstrated that one single electron at an energy near 2 eV can induce the excision of CN^−^ from acetamide, which proceeds by a complex reaction associated with the concerted cleavage of several bonds and the formation of new molecules (such as H_2_O).[5] In theoretical studies, it has been predicted that a captured LEE can simultaneously break up to four bonds in cyclic compounds before the excess electron is liberated again.[6,7] Herein we study the attachment of LEEs to 4-nitroimidazole (4NI) und 1-methyl-4-nitroimidazole (Me4NI) (Figure 1) by means of a crossed electron-molecular beams experiment and mass spectrometric detection of the anions formed. We find that both molecules show a remarkably high sensitivity towards LEEs in the energy range 0–6 eV in the way that a variety of fragment anions are observed arising from unimolecular decomposition reactions. While both compounds generate identical final ionic products (except for H-loss as expected), we find that at electron energies below 2 eV their intensity is completely suppressed in Me4NI, that is, upon methylation of 4NI at the N1 site. This effect is interpreted by the formation of vibrational Feshbach resonances (VFRs) which couple with dissociative states leading to the various DEA fragments. VFRs are electronic states of the anion in which the excess electron is weakly bound in the field of the corresponding vibrationally excited molecule.[8]

**Figure 1:**
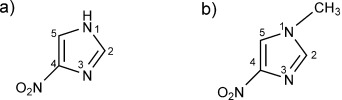
Molecular structures of a) 4-nitroimidazole (4NI), b) 1-methyl-4-nitroimidazole (Me4NI).

The nitroimidazolic compounds are presently under investigation as potential radiosensitizers for so called hypoxic tumors, which are tumors deprived of oxygen.[9] Such low-oxygen states are known as hypoxia. As in the description of radiotherapy at a molecular level the reactions induced by slow secondary electrons may play a significant role,[2,10] it is of immediate interest to study the response of these potential radiosensitizers towards LEEs.

The experiments are performed by means of an electron attachment spectrometer consisting of a molecular beam source, an electron monochromator (HEM), and a quadrupole mass filter for analyzing and detecting the ions formed.[11] The 4NI sample was purchased from Sigma–Aldrich with stated purity of 97 %. The Me4NI sample had a purity of 98 % and was purchased from BOC-Sciences, respectively. Both were evaporated in an oven inside the vacuum chamber and the resulting vapors were introduced into the interaction chamber via a capillary. The oven temperatures used were about 373 K for 4NI and 333 K for Me4NI. The HEM was operated at an energy resolution of 110–150 meV (FWHM) and at an electron current of 20 nA for a reasonable compromise between resolution and ion intensity.

In the following we shall demonstrate that the rich chemistry in 4NI induced by slow electrons is completely suppressed in the energy range below 2 eV in its methylated form (Me4NI). This not only concerns the loss of a neutral hydrogen atom via DEA but also bond cleavages and reactions involving the entire molecular system. This significant and surprising effect is demonstrated for a) the loss of a neutral hydrogen atom; b) cleavage of the C=NO_2_ bond; and c) a more complex unimolecular reaction leading to the loss of a neutral ^.^OH unit.

Figure 2 (top) shows the ion yield of the largest fragment ion observed from nitroimidazole (4NI) at 112 u, which arises from the loss of a neutral hydrogen atom according to the DEA reaction (reaction (2)):

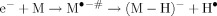
(2)

**Figure 2:**
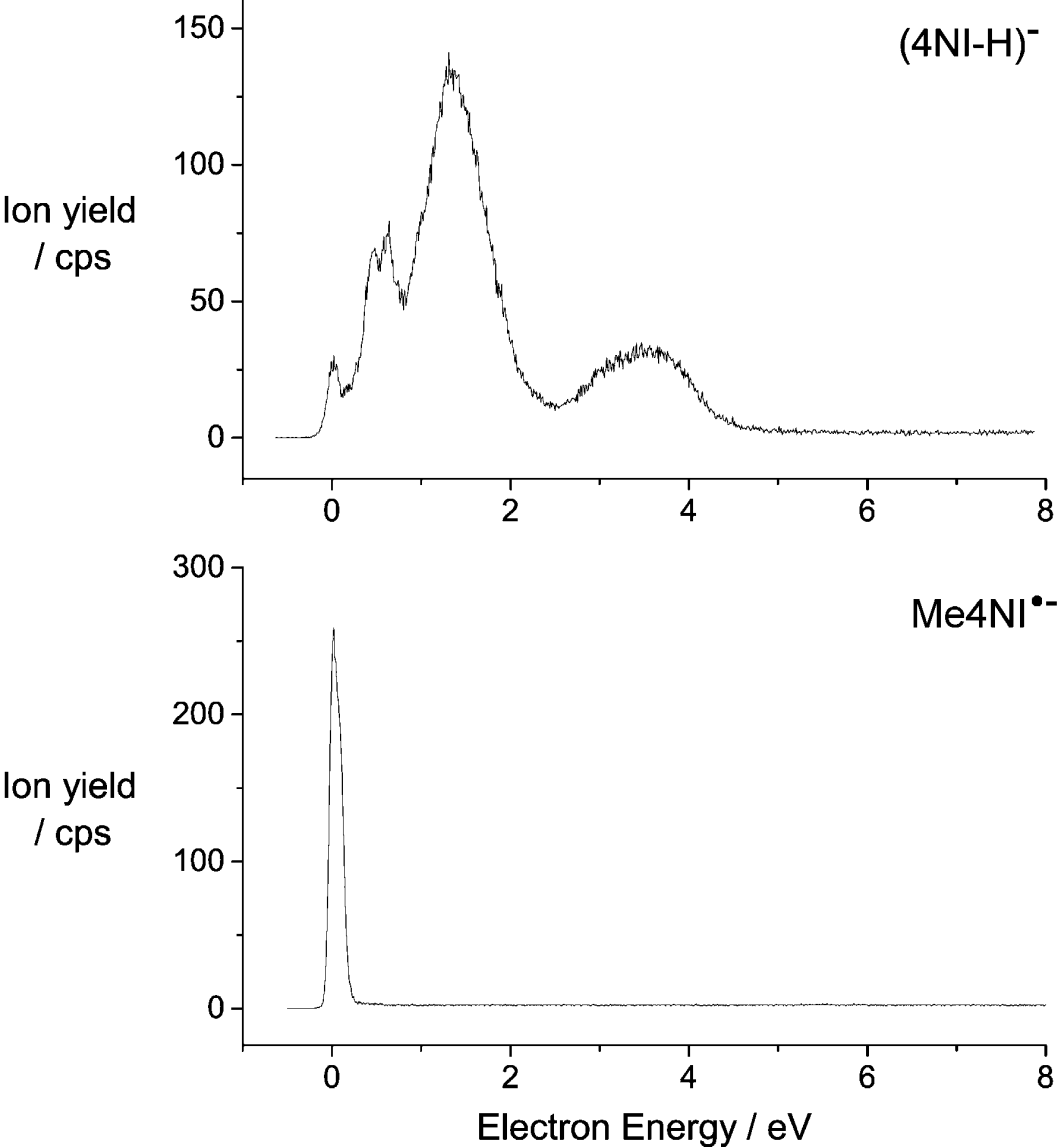
Relative cross-section for the formation of (M−H)^−^ from 4NI (top) and the metastable parent anion M^.−^ in Me4NI (bottom).

with M^.−#^ the transient intermediate formed upon electron attachment and (M−H)^−^ the closed shell dehydrogenated anion. The reaction enthalpy for DEA reaction (2) amounts to 0.39 eV,[12] which matches well with the experiment. The small zero eV contribution is formed upon DEA to 4NI thermally excited by the evaporation process. No signal is detected from Me4NI at 126 u, which indicates that in 4NI the loss of H exclusively occurs from the N1 site and that in Me4NI this dissociation channel is blocked as expected.

The yield of the dehydrogenated ion (M−H)^−^ exhibits an interesting series of sharp peaks below 2 eV and a broader contribution peaking at 3.4 eV. Sharp peaks at very low energies are often interpreted as VFRs,[8] and consequently we assign these features to VFRs. 4NI and Me4NI have overcritical dipole moments of 7.78 and 8.50 Debye (calculated at M062x/6-311+G(d,p) level of theory), respectively, which further supports this assignment.

A qualitatively similar behavior was recently observed in the DNA base thymine (T), where the loss of H exclusively involves the two N sites,[3] and where in the yield of (M−H)^−^ sharp peaks in the energy below 1.2 eV were observed followed by an unstructured broader distribution peaking at 1.8 eV.

As the basic DEA mechanism proposed in T most likely also applies for 4NI and Me4NI, it will be briefly considered in the following. It has to be noted that in T the only reaction operative at energies below 3 eV is hydrogen loss, while 4NI shows a much richer chemistry.

Methylation of T at the N1 site resulted in a complete quenching of the sharp structures below 1 eV and thus they were associated with DEA, leading to the loss of H exclusively from the N1 site.[4] A detailed analysis identified these structures as VFRs dominantly involving the N1=H stretch vibration.[11,13] These VFRs couple to the repulsive σ* (N1=H) configuration ultimately leading to hydrogen loss from the N1 site. The unstructured contribution at 1.8 eV (dominantly associated with the loss of H from the N3 position) is formed by electron attachment into a π* MO and vibronic coupling, that is, a mixing of states with repulsive σ* valence character through vibrational motion along the dissociating bond.

The present situation is apparently very similar to that in T, and as already considered above we assign the series of sharp peaks below 2 eV as VFRs involving the N1=H stretch and eventually further vibrational modes. However, we also note that the adiabatic electron affinity of 4NI is 0.73 eV,[12] that is, vibrationally excited valence anions may also form, which may be coupled with the dipole bound states, as previously proposed for the nitromethane anion.[14] At that point, we resume from a more detailed analysis of these structures which is not necessary for the discussion of the significant effects presented here. The unstructured resonance peaking at 3.4 eV is then assigned as π* resonance subjected to vibronic coupling.

As mentioned, no H-loss is detected for Me4NI. Instead, Me4NI does show the appearance of a metastable parent anion (M^.−^, 127 u) right at threshold (Figure 2, bottom). The fact that in Me4NI a parent anion is observed (which is not the case in 4NI) will not be discussed in any detail here. We only mention that in this case an efficient transfer of the energy of the incoming electron to the internal degrees of freedom of the target molecule is required in order to delay autodetachment (reaction (1 b)) into the μs time regime.[15]

Figure 3 shows the spectra arising from the cleavage of the C-NO_2_ bond, which proceeds through two complementary DEA reactions:3a, 3b

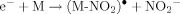
(3a)

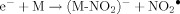
(3b)

**Figure 3:**
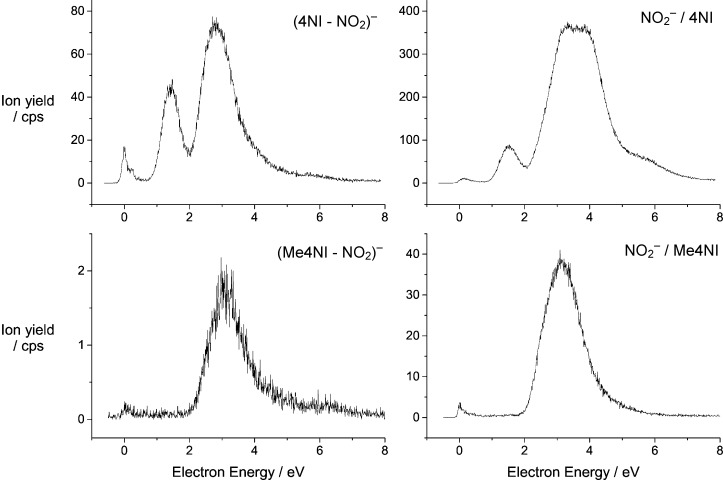
Relative cross-sections for the formation of the complementary ions NO_2_^−^ and (M−NO_2_)^−^ from 4NI and Me4NI.

Obviously for both of these complementary ions NO_2_^−^ and (M-NO_2_)^−^ the reaction below 2 eV is completely suppressed in Me4NI. The small peak right at threshold (0 eV) is most likely an artefact and reminiscent to the peculiarities of DEA.[1] The reaction enthalpy (Δ*H*°) for the DEA reactions (3a) and (3b) is given by the D(C=NO_2_) bond dissociation energy minus the electron affinity of the fragment on which the excess electron finally becomes localized. For reaction (3a), Δ*H*° is 0.8 eV obtained from the established average bond dissociation energy in aromatic nitro compounds (D(C=NO_2_)=3.1 eV[16]) and the electron affinity of NO_2_ (2.27 eV).[17] This example demonstrates that methylation at N1 not only blocks the cleavage of the N1=CH_3_ bond by LEEs but also that of the C4=NO_2_ bond.

The last example presented herein concerns an appreciably complex DEA reaction, namely the loss of a neutral OH unit resulting in an ion detected at 96 u from 4NI and at 110 u from Me4NI (Figure 4). This reaction requires the cleavage of two bonds and the formation of OH. Again, the reaction is suppressed in Me4NI in the energy domain below 2 eV, indicating that Feshbach resonances present in 4NI couple to valence configurations associated with multiple bond cleavages. A closer inspection of the energy range between 0 and 2 eV reveals that the presence and relative intensity of the sharp peaks slightly varies between the different ionic products under observation. The overlapping sharp peaks at 0.7 and 0.8 eV observed in the relative DEA cross-section of (M−H)^−^ are not present in the cross-section of the other fragment ions.

**Figure 4:**
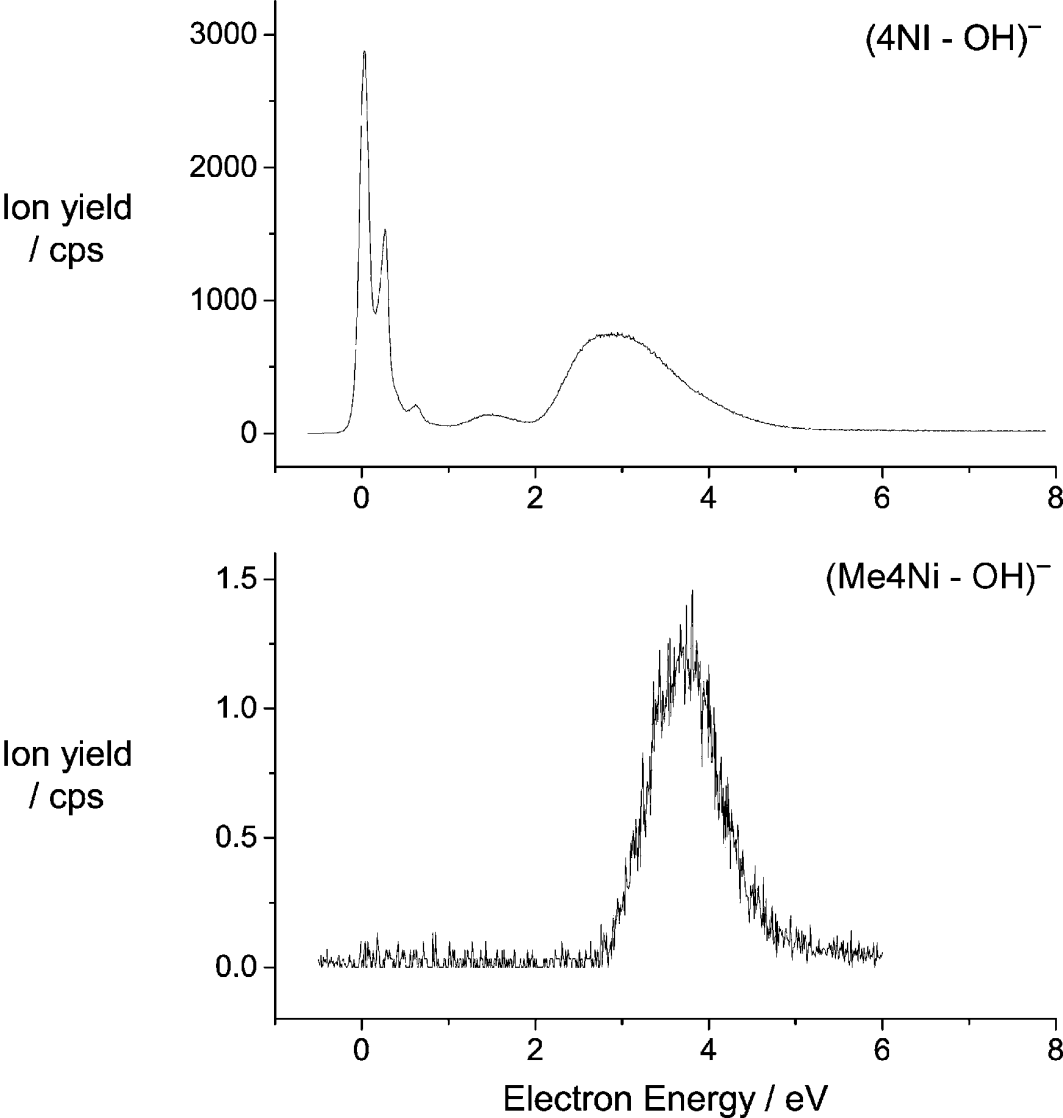
Relative cross-section for the formation of the ion arising from the loss of a neutral ^.^OH radical ((M−OH)^−^) from 4NI and Me4NI.

Along the same lines, for both compounds we observe an ion arising from the loss of a neutral CO_2_ unit ((M-CO_2_)^−^), and the formation of CN^−^ and OCN^−^ (not shown here). While CN^−^ can be formed by the excision of two adjacent atoms C and N, the formation of OCN^−^ additionally requires transfer of an O atom. In all of these cases, the corresponding ion yields show sharp structures in the energy range below 2 eV in 4NI which are completely suppressed in Me4NI. These examples further show, that methylation at the N1 site affects the entire molecule and that the VFRs act as effective doorways for a variety of DEA reactions. We also note that it is irrelevant on which C-position the NO_2_ unit is coupled. In fact, DEA experiments on the isomer 1-methyl-5-nitroimidazole (M5NI) revealed the same fragment ions and virtually identical resonance positions compared to Me4NI.[18]

In conclusion, it is shown that in nitroimidazole the sharp structures present in the relative partial DEA cross-sections in the energy range below 2 eV are completely suppressed upon methylation on the N1 site. Most remarkably, methylation not only blocks the cleavage of the N1=CH_3_ bond, but suppresses the entire rich chemistry of the molecule in the energy range below 2 eV. With respect to the potential use as radiosensitizers, it can only be stated that in nitroimidazole, secondary LEEs with energies 0–2 eV formed by ionizing radiation generate radicals and among them the ^.^OH radical. The DNA damage by these radicals represents the so-called indirect DNA damage, which is estimated to contribute up to two thirds to the DNA damage induced by ionizing radiation.[2,10]
